# Condy’s Fluid and Carbolic Acid

**Published:** 1866-12

**Authors:** 


### Condy's Fluid and Carbolic Acid.

All London is now smelling of carbolic acid. Placards recommending disinfectants are affixed to every wall, and in the parish in which we live men are going round the houses of the poor with instructions to put a dose of carbolic acid into every sink and closet, and to put half an ounce of Condy's red fluid into every water receptacle that is made of wood. These measures are taken in the belief that something dangerous lurks in dirty drains which carbolic acid can destroy, and something in suspicious drinking water which Condy's fluid can destroy, leaving the water fit for use. So far as regards cholera, the dangerous matter may be of three kinds: either living matter of some low sort, as held by Dr. Beale, and rendered most highly probable by the Cattle Plague Reports; or an alkaloid, as held by Dr. Richardson; or, lastly, matter in a state of change, according to Liebig's theory, which last hypothesis is not inconsistent with the first.

Condy's fluid is a solution of some permanganate; for our present purpose let us say permanganate of potass. One equivalent of this salt = 158 is calculated to lose one-fourth of its weight of oxygen in presence of oxidisable matter, and in so doing loses the pink transparency of its solution, and forms a brown precipitate. The quantity of oxidisable matter may either be estimated by giving the quantity of oxygen in the decolorised permanganate simply, or on Dr. Letheby's method, by multiplying the amount of oxygen by 8. But we cannot at present go into the process, which will be found well described in the papers above referred to. We want rather to come to one or two practical points of present application.

Let us suppose a water of a bad, or at least a suspicious marshy smell; the addition of one or more drops of "Condy," or of one of the finer solutions of permanganate, will speedily remove that smell and taste, and make the water fresher and nicer. The quicker the decolorisation, the greater the need of it.

If water so treated, with a slight pink color remaining, be passed through a filter, it comes out perfectly clear and colorless; but without filtering may be used for cooking or making tea and coffee after the brown sediment has settled. Most assuredly any one thirsty enough to drink raw London water just now had better use the permanganate and filter too.

It seems generally agreed that the gases of decomposition are very quickly neutralised by this means, and that organic matter actually decomposing very quickly decolorises the liquid also. But this is not the case with stable organic matter. Water colored with Condy so as not to be drinkable with pleasure, yet may contain animalcules in the most lively state. Nay, the amœba, paramœcium, colpods, and other disgusting broods are not in the least affected by water too reddened to be drinkable. The same with regard to minute plants. Give quantity enough and time enough, and all will be destroyed—first, the stinking gases; next, the decaying organic matter which evolves them; then the microscopic animalcules which feed on it, and which, if not destroyed by the Condy, would die of starvation; and the plants last.

Time and quantity also are required for the destruction of such a substance as the bitter extract which is diffused into water from quassia; this may bc got rid of in twelve hours. The resistance of strychnia is much greater; still a very weak solution may be deprived of all bitter taste by excess of permanganate in twenty-four hours. Matters having organic form and firmness, as starch, etc., if not decomposing, are very slowly acted on.

Animalcules of the kinds indicated may also live in water just containing carbolic acid enough to be smelt and tasted.

The conclusions we would draw from the above remarks are that when we employ the carbolic acid for the disinfection of drains, sinks, etc., it onght to be employed in a state of pretty high concentration and large quantity, so as, above all things, to purify the aperture out of which the dangerous emanations would come. Likewise in the use of Condy's fluid for purifying water-butts, enough should be used, but wc should take care also that the butts themselves are cleansed, and pitched or charred inside, for it is a waste of force to use the permanganate to do what might be done by a handful of lighted shavings and a brimstone match.

We may add that at least one traveler of our acquaintance used the permanganate daily for some time with no ill effects whatever. [Med. Times and Gazette, Aug. 11, 1866.

